# High Lipoprotein(a) Level Is Independently Associated with Adverse Clinicopathological Features in Patients with Prostate Cancer

**DOI:** 10.1155/2019/9483935

**Published:** 2019-11-22

**Authors:** Fang-Ming Wang, Yan Zhang

**Affiliations:** ^1^Department of Urology, The Affiliated Hospital of Qingdao University, Qingdao, Shandong 266003, China; ^2^Division of Dyslipidemia, State Key Laboratory of Cardiovascular Disease, Fu Wai Hospital, National Center for Cardiovascular Diseases, Chinese Academy of Medical Sciences, Peking Union Medical College, Beilishi Road 167, Beijing 100037, China

## Abstract

**Background:**

The effect of lipoprotein(a) (Lp(a)) on prostate cancer (PCa) is unclear. The aim of this study was to investigate the association between serum Lp(a) levels and clinicopathological features in patients with PCa.

**Methods:**

A total of 376 consecutive pathologically diagnosed PCa patients were enrolled and were classified as a low-intermediate-risk group or a high-risk group. The association of Lp(a) and the other lipid parameters including total cholesterol (TC), low-density lipoprotein cholesterol (LDL-C), high-density lipoprotein cholesterol (HDL-C), triglycerides (TG), TC/HDL-C, LDL-C/HDL-C, and remnant cholesterol (RC) with clinicopathological parameters was tested by univariate and multivariate logistic regression analyses.

**Results:**

The high-risk PCa patients tended to have higher Lp(a) levels (*p* = 0.022) while there was no significant difference regarding the other lipid parameters (*p* > 0.05) compared to low-intermediate-risk counterparts. Patients with PSA ≥ 100 ng/ml had significantly higher Lp(a) levels than subjects with PSA < 100 ng/ml (*p* = 0.002). Univariate logistic regression analyses revealed that high Lp(a) levels were correlated with high-risk PCa (Q4 vs. Q1, HR = 2.687, 95% CI: 1.113-6.491, *p* = 0.028), while the other lipid parameters were not correlated with high-risk PCa. In the stepwise multivariate regression analysis, the association between Lp(a) levels and high-risk PCa remained significant (Q4 vs. Q1, HR = 2.890, 95% CI: 1.148-7.274, *p* = 0.024) after adjusting for confounding factors including age, body mass index, hypertension, diabetes, coronary artery disease, and lipid-lowering drugs.

**Conclusions:**

This is the first study showing the positive association between high Lp(a) and adverse clinicopathological features of PCa. PCa patients with high Lp(a) tends to be more aggressive and should receive more attention in clinical practice.

## 1. Introduction

Lipoprotein(a) (Lp(a)) is a lipoprotein synthesized by the liver, consisting of a low-density lipoprotein (LDL) core with an apolipoprotein B-100 molecule covalently linked to apolipoprotein(a) (apo(a)) [1]. Lp(a) levels are essentially genetically determined and rather stable over time; they vary widely between individuals and show right-skewed distribution in the population. Besides, Lp(a) levels are less influenced by diet or lipid-lowering drugs [2] but are closely related to female sex hormones and increased after the menopause [3]. Large-scale prospective cohort studies have shown that high plasma concentration of Lp(a) is a risk factor for cardiovascular diseases (CVD) and stroke [4–7]. Recently, increased attention has been paid to the effect of Lp(a) on tumors. Till now, some experimental studies support the antineoplastic effect of apo(a) or Lp(a) [8, 9], but some clinical studies have reported contradictory results, demonstrating that higher cancer risk was observed for the highest Lp(a) levels in lung and colorectal cancers [10, 11].

To date, few studies have investigated potential associations between Lp(a) and the risk of prostate cancer (PCa). A recent study revealed that higher levels of Lp(a) were associated with an increase in PCa incidence risk [12]. Another study demonstrated that the lowest risk of PCa was observed for the highest levels of Lp(a) [11].

However, to the best of our knowledge, whether Lp(a) is associated with the clinicopathological characteristics of PCa has not been reported. Accordingly, in the present study, we sought to determine the association between Lp(a) and clinicopathological features including the risk severity of PCa in 376 pathologically diagnosed patients.

## 2. Material and Methods

### 2.1. Study Design and Population

We followed our previous methods [13]. The study was performed in accordance with the Declaration of Helsinki and was approved by the ethical committee of the Affiliated Hospital of Qingdao University. All subject names, initials, or hospital numbers were not used in the text, table, or illustrative materials of this study.

The study was conducted in patients with primary diagnosed, pathologically confirmed sporadic PCa, between January 2011 and October 2018 at the Department of Urology at the Affiliated Hospital of Qingdao University. All patients were Chinese Han people. The exclusion criteria of the study were the presence of medical history of other malignancies, severe liver and/or renal insufficiency, or incomplete clinicopathological information. All data on age, body mass index (BMI), history of hypertension, diabetes, coronary artery disease (CAD), lipid-lowering drugs, serum PSA, cancer grade, tumor clinical stage at diagnosis, treatment protocols, and lipid profiles were obtained from electronic records and medical charts. All the pathological data analyzed in this study were identified from the transrectal ultrasound-guided prostate biopsy or radical prostatectomy specimens. All specimens were processed according to standard pathological procedures. The tumor stage was assessed according to the American Joint Committee on Cancer (AJCC) TNM classification of malignant tumors 2002. Cancer grade was assessed according to the ISUP classification of 2014 [14].

### 2.2. Blood Sampling and Lipoprotein(a) Measurement

Venous blood samples were collected from all subjects after overnight fasting according to a standardized protocol. Blood samples were obtained from PCa patients before surgical management, androgen deprivation, or other therapies. Lp(a) levels were measured by the turbidimetric immunoassay method (LASAY Lp(a) auto; SHIMA Laboratories Co., Ltd.) with a normal value of <30 mg/dl. An Lp(a) protein validated standard was used to calibrate the examination, and the coefficient of variation value of repetitive measurements was <10%. Total cholesterol (TC), high-density lipoprotein cholesterol (HDL-C), low-density lipoprotein cholesterol (LDL-C), and triglycerides (TG) were determined using enzymatic methods on an Hitachi 7600D clinical chemistry analyzer according to the manufacturer's instructions. Remnant cholesterol (RC) was calculated by TC − HDL − LDL.

### 2.3. Statistical Analyses

Quantitative variables were expressed as mean ± standard deviation (SD) except for Lp(a) and TG, which were expressed as median with interquartile range, and were analyzed by Student's *t*-tests, one-way ANOVA, Mann-Whitney *U* tests, or Kruskal-Wallis tests as appropriate. The qualitative variables were expressed as numbers and percentages and were analyzed by chi-squared statistic tests. All PCa subjects were divided into two groups: low-intermediate-risk group and high-risk group according to the PSA, Gleason score, and clinical stage. Low-intermediate risk is defined as follows: PSA ≤ 20 ng/ml, ISUP grade ≤ 3, and clinical stage ≤ T2b; high risk was based on either one of the following criteria: PSA > 20 ng/ml, ISUP grade ≥ 4, or clinical stage ≥ T2c. The stratification of PCa risk was conducted by two surgeons, and they were blind to Lp(a) levels for these same patients. Correlations between variables with high-risk PCa were examined by univariate logistic regression analysis. Stepwise multivariate regression analysis was used to determine the independent factors of high-risk PCa with adjustment for potential confounding factors including age, BMI, hypertension, diabetes, CAD, and lipid-lowering drugs.

Besides, we divided Lp(a) into four quartiles as follows: the first quartile group (Q1, <104 mg/l (25th percentile); *n* = 95), the second quartile group (Q2, 104-216 mg/l (25-50th percentile); *n* = 93), the third quartile group (Q3, 216-381 mg/l (50-75th percentile); *n* = 94), and the fourth quartile group (Q4, >381 mg/l (75-100th percentile); *n* = 94), and analyzed clinicopathological features in different levels of Lp(a). A *p* value of less than 0.05 was considered statistically significant. Statistical studies were carried out with the SPSS program (version 19.0, SPSS, Chicago, Illinois, USA).

## 3. Results

### 3.1. Baseline Characteristics

The current study consisted of 376 eligible pathologically confirmed PCa patients (male, with a mean age of 70.8 ± 7.9 years), including 54 cases of low-intermediate-risk and 322 cases of high-risk subjects.

The baseline demographic, clinical characteristics, and laboratory findings of the enrolled subjects are summarized in Table 1. In brief, no significant differences in age, BMI, hypertension, diabetes, CAD, and lipid-lowering drugs were observed between low-intermediate- and high-risk groups.

Lp(a) levels of the high-risk group were significantly higher than those of the low-intermediate-risk group (224 (110-401) versus 165 (71-334), *p* = 0.022). However, there was no significant difference regarding TG, TC, LDL-C, HDL-C, TC/HDL-C, LDL-C/HDL-C, or RC between low-intermediate- and high-risk groups (*p* > 0.05).

To evaluate the lipid characteristics according to PSA levels, we classified all subjects into two groups: PSA < 100 ng/ml (*n* = 220) and PSA ≥ 100 ng/ml (*n* = 156). As shown in Table 2, we observed that patients with PSA ≥ 100 ng/ml had a significantly higher Lp(a) level but a lower BMI compared to those with PSA < 100 ng/ml (253.0 (139.3-445.8) ng/ml versus 188 (90-359) ng/ml, *p* = 0.002; 23.8 ± 3.1 versus 25.0 ± 3.5, *p* = 0.001, respectively). There was no significant difference regarding TG, TC, LDL-C, HDL-C, TC/HDL-C, LDL-C/HDL-C, or RC (*p* = 0.888, *p* = 0.180, *p* = 0.140, *p* = 0.099, *p* = 0.764, *p* = 0.919, and *p* = 0.279, respectively).

### 3.2. Clinicopathological Characteristics in Different Lp(a) Levels

PCa patients were divided into four groups in terms of serum Lp(a) level quartiles as mentioned above. Statistically significant differences in BMI, hypertension, stage, and metastasis were found among different groups (*p* = 0.001, *p* = 0.015, *p* = 0.017, and *p* = 0.018, respectively). Specifically, the percentage of PCa metastasis increased gradually with the elevation of Lp(a) level and was highest in Q3 and Q4, a statistically significant difference compared with the other groups (*p* = 0.014), while BMI decreased with the elevation of Lp(a) level (*p* = 0.011). There is an evident trend that PSA ≥ 100 ng/ml and lymph node involvement percentage rose with the elevation of Lp(a) level although the difference was not statistically significant (*p* = 0.075, *p* = 0.137). There were no differences in age, diabetes, CAD, or ISUP grade among the groups (Table 3).

### 3.3. Correlations of Lp(a) and the Other Lipid Parameters with High-Risk PCa

To evaluate the correlations of Lp(a) and other lipid parameters with high-risk PCa, univariate logistic regression analysis was performed in the current study. As shown in Table 4, Lp(a) found to be statistically significant in univariate analyses was entered into multivariate logistic regression analysis. The data indicated that the high Lp(a) level was still independently correlated with the presence of high-risk PCa after adjusting for confounders including age, BMI, hypertension, diabetes, CAD, and lipid-lowering drugs in multivariate logistic regression analysis (Q4 vs. Q1, OR = 2.890, 95% CI: 1.148–7.274, *p* = 0.024) (Table 4). Nevertheless, we did not observe a statistically significant relationship between high-risk PCa and other lipid parameters including TG, TC, LDL-C, HDL-C, TC/HDL-C, LDL-C/HDL-C, and RC (Table 4).

## 4. Discussion

The present study evaluated the association between high Lp(a) and adverse clinicopathological features of PCa. To our knowledge, this is the first study to demonstrate that patients with high Lp(a) tended to have higher risk of aggressive PCa compared with those with low Lp(a). This association remained significant after adjusting for other confounders. Therefore, the current study might have potential clinical implications with regard to the assessment of PCa risk based on the lipid perspectives. For example, for one PCa patient with high Lp(a), he should be followed up more closely and receive more attention in clinical practice. Besides, our study provided novel information to explore more possible mechanisms of PCa.

There is no doubt that circulating levels of Lp(a) and other lipid parameters are associated with CVD and are considered major indicators of metabolic health [6, 12, 15]. Recently, more and more research has focused on the relationship between Lp(a) and multiple kinds of tumors. Results from different studies are not entirely consistent. It is reported that patients with lung or breast cancer exhibit elevated Lp(a) levels [10, 16], while the Lp(a) level was relatively low in patients with hepatocellular carcinoma [17]. Marrer et al. [11] conducted a cohort study analyzing cancer incidence in relation to Lp(a) plasma levels and found that men with the highest Lp(a) levels seemed to have the highest risk of all-site, lung, colorectal, or tobacco/alcohol-related cancers, but the lowest risk of PCa. One could expect that if Lp(a) levels increase the incidence of cancer, they would also increase cancer mortality. However, a Japanese cohort study analyzing the association between Lp(a) levels and cancer deaths found an increased risk for low Lp(a) levels, when comparing the lowest quartile versus the three upper quartiles combined [18].

However, few studies on the relationship between Lp(a) and PCa have been reported. We reviewed the literatures and found that Katzke and colleagues [12] had reported that high Lp(a) levels were significantly associated with a 1.5 higher risk of incident PCa (Q4 vs. Q1, HR = 1.47). On the contrary, as mentioned above, Marrer et al. [11] demonstrated that the lowest risk of PCa was observed for the highest levels of Lp(a). Most studies compared the PCa patients with normal people and did not reveal the relationship between Lp(a) and risk of aggressiveness or invasion within patients with PCa.

Our study divided PCa patients into two groups: low-intermediate-risk group and high-risk group, then analyzed lipid parameters in the two groups, and found that Lp(a) was the only lipid parameter that differs between the two groups. Next, we demonstrated, by both univariate and multivariate analyses, that high Lp(a) independently correlated with the presence of high-risk PCa. Besides, we found that BMI was significantly lower in the high-Lp(a) group and PSA ≥ 100 ng/ml group than in the low-Lp(a) group and PSA < 100 ng/ml group, which could be explained by the excessive energy consumption and weight loss in advanced PCa. Although there were no significant differences on positive pelvic lymph node between individuals with different levels of Lp(a), we can see a trend that patients with high Lp(a) (Q3 and Q4) had higher risk of lymph node metastasis, which was consistent with the significant result of bone metastasis.

There lack studies focusing on the relationship of Lp(a) and clinicopathological features in cancers. Our findings are similar to one recent study [19] in liver cancer, which revealed that hepatocellular carcinoma patients with portal vein thrombosis showed a statistical significant serum Lp(a) level higher than those without portal vein thrombosis.

The potential mechanisms of this correlation of high Lp(a) and adverse clinicopathological features of PCa remain uncertain and need further studies. Lp(a) plasma levels are genetically determined, affected only to a minor extent by age, sex, and environmental factors, and rather stable in individuals [2, 20]. We speculate that there are two main completely different mechanisms to explain our study results. Firstly, some studies on animal models have indicated that the proteolytic breakdown products of Lp(a) possess antitumoral properties both in vitro and in vivo [21–23]. Considering the antitumoral role of Lp(a), we believe that the elevated Lp(a) levels in high-risk and PSA ≥ 100 PCa patients might be compensatory reactions to chronic inflammation of the whole body caused by aggressiveness and invasion of a tumor. Secondly, apo(a), as an important component of Lp(a), is essentially composed of structural homologues to kringle IV of the plasminogen molecule and functions as a competitive inhibitor for the activation of plasmin-induced fibrinolysis [24, 25]. Therefore, high Lp(a) could more easily induce the formation of fibrin network and thrombus, facilitating cancer cell adhesion, invasion, and metastasis. Moreover, hormonal imbalance may affect Lp(a) levels in the context of prostate cancer.

There were several limitations in our study. Firstly, it was conducted at a single center. Secondly, this was an observational study and could not identify the causal relationship. Finally, we did not evaluate the prognostic value of Lp(a) in the present study.

## 5. Conclusions

In conclusion, this study provided evidence of an association between Lp(a) and clinicopathological features of PCa: patients with high Lp(a) had more adverse clinicopathological features of PCa than those with low Lp(a). This association remained significant after adjustment for other confounding factors. Further basic research on PCa genetic or biological differences associated with Lp(a) is needed in the future studies.

## Figures and Tables

**Table 1:** Baseline characteristics of the study subjects according to the risk of PCa.

	All subjects (*n* = 376)	Low-intermediate risk (*n* = 54)	High risk (*n* = 322)	*p* value
Demographic characteristics				
Age (years)	70.8 ± 7.9	69.8 ± 5.8	70.9 ± 8.2	0.230
BMI (kg/m^2^)	24.5 ± 3.4	24.7 ± 3.4	24.4 ± 3.4	0.639
Hypertension (*n* (%))	140 (37.2)	19 (35.2)	121 (37.6)	0.764
Diabetes mellitus (*n* (%))	49 (13.0)	10 (18.5)	39 (12.1)	0.273
Coronary artery disease (*n* (%))	56 (14.9)	6 (11.1)	50 (15.5)	0.424
Lipid-lowering drugs (*n* (%))	9 (2.4)	2 (3.7)	7 (2.2)	0.623
Lipid parameters				
TG (mmol/l)	1.04 (0.76-1.39)	1.07 (0.68-1.49)	1.04 (0.77-1.38)	0.520
TC (mmol/l)	4.85 ± 0.97	4.91 ± 0.91	4.84 ± 0.98	0.634
LDL-C (mmol/l)	2.79 ± 0.75	2.88 ± 0.75	2.78 ± 0.75	0.359
HDL-C (mmol/l)	1.35 ± 0.31	1.34 ± 0.34	1.36 ± 0.30	0.714
TC/HDL-C	3.69 ± 0.88	3.83 ± 0.98	3.67 ± 0.87	0.213
LDL-C/HDL-C	2.15 ± 0.69	2.27 ± 0.75	2.12 ± 0.68	0.141
RC (mmol/l)	0.71 ± 0.34	0.70 ± 0.33	0.71 ± 0.35	0.903
Lp(a) (mg/l)	216 (104-382)	165 (71-334)	224 (110-401)	**0.022**
Surgery (*n* (%))				
Radical prostatectomy	75 (19.9)	26 (48.1)	49 (15.2)	<**0.001**
Bilateral orchiectomy	55 (14.4)	0	54 (16.8)	**0.001**

Data are expressed as *n* (%), mean ± SD, or median (25th-75th percentile). The bold value indicated statistical significance. PCa = prostate cancer; BMI = body mass index; PSA = prostate-specific antigen; RC = remnant cholesterol; TC = total cholesterol; HDL-C = high-density lipoprotein cholesterol; LDL-C = low-density lipoprotein cholesterol; TG = triglycerides; Lp(a) = lipoprotein(a).

**Table 2:** Lipid parameters of the study population stratified by PSA values.

	PSA values		
	PSA < 100 (*n* = 220)	PSA ≥ 100 (*n* = 156)	*p* value
Demographic characteristics			
Age (years)	71.4 ± 7.7	70.0 ± 8.2	0.071
BMI (kg/m^2^)	25.0 ± 3.5	23.8 ± 3.1	**0.001**
Hypertension (*n* (%))	85 (38.6)	54 (34.6)	0.448
Diabetes mellitus (*n* (%))	31 (14.1)	18 (11.5)	0.535
Coronary artery disease (*n* (%))	39 (17.7)	17 (10.9)	0.078
Lipid-lowering drugs (*n* (%))	7 (3.2)	2 (1.3)	0.315
Lipid parameters			
TG (mmol/l)	1.04 (0.73-1.39)	1.05 (0.78-1.39)	0.888
TC (mmol/l)	4.90 ± 0.95	4.77 ± 1.00	0.180
LDL-C (mmol/l)	2.84 ± 0.75	2.72 ± 0.76	0.140
HDL-C (mmol/l)	1.38 ± 0.32	1.32 ± 0.29	0.099
TC/HDL-C	3.68 ± 0.88	3.71 ± 0.90	0.764
LDL-C/HDL-C	2.15 ± 0.68	2.14 ± 0.71	0.919
RC (mmol/l)	0.69 ± 0.33	0.73 ± 0.36	0.279
Lp(a) (mg/l)	188 (90-359)	253.0 (139.3-445.8)	**0.002**

Data are expressed as *n* (%), mean ± SD, or median (25th-75th percentile). The bold value indicated statistical significance. PCa = prostate cancer; BMI = body mass index; PSA = prostate-specific antigen; RC = remnant cholesterol; TC = total cholesterol; HDL-C = high-density lipoprotein cholesterol; LDL-C = low-density lipoprotein cholesterol; TG = triglycerides; Lp(a) = lipoprotein(a).

**Table 3:** Demographic and clinicopathological characteristics of study subjects according to the quartiles of Lp(a) plasma levels.

	Quartiles of Lp(a)				
	<104 (*n* = 95)	104-216 (*n* = 93)	216-381 (*n* = 94)	>381 (*n* = 94)	*p* value
Demographic characteristics					
Age (years)	71.3 ± 8.3	70.4 ± 7.3	70.2 ± 8.9	71.2 ± 7.1	0.668
BMI (kg/m^2^)	25.4 ± 3.3	24.8 ± 3.2	24.1 ± 3.5	23.4 ± 3.3	**0.001**
Hypertension (*n* (%))	48 (50.5)	30 (32.3)	34 (36.2)	28 (29.8)	**0.015**
Diabetes mellitus (*n* (%))	11 (11.6)	11 (11.8)	9 (9.6)	18 (19.1)	0.222
Coronary artery disease (*n* (%))	14 (14.7)	13 (14.0)	15 (16.0)	14 (14.9)	0.989
Lipid-lowering drugs (*n* (%))	4 (4.2)	3 (3.2)	1 (1.1)	1 (1.1)	0.424
Clinicopathological characteristics					
PSA ≥ 100 (*n* (%))	29 (30.5)	39 (42.4)	44 (46.8)	44 (46.8)	0.075
ISUP grade (*n* (%))					0.837
1	14 (14.7)	7 (7.5)	11 (11.7)	9 (9.6)	
2	7 (7.4)	7 (7.5)	6 (6.4)	10 (10.6)	
3	13 (13.7)	11 (11.8)	12 (12.8)	12 (12.8)	
4	25 (26.3)	24 (25.8)	27 (28.7)	18 (19.1)	
5	36 (37.9)	44 (47.3)	38 (40.4)	45 (47.9)	
Stage (*n* (%))					**0.017**
T1	7 (7.4)	3 (3.2)	3 (3.2)	2 (2.1)	
T2	57 (60.0)	44 (47.3)	47 (50.0)	57 (60.6)	
T3	24 (25.3)	35 (37.6)	22 (23.4)	22 (23.4)	
T4	7 (7.4)	11 (11.8)	22 (23.4)	13 (13.8)	
N1	23 (24.2)	28 (30.1)	37 (39.4)	33 (35.1)	0.137
M1	27 (28.4)	41 (44.1)	45 (47.9)	45 (47.9)	**0.018**

Data are expressed as *n* (%), mean ± SD, or median (25th-75th percentile). The bold value indicated statistical significance. PCa = prostate cancer; BMI = body mass index; PSA = prostate-specific antigen; ISUP = International Society of Urological Pathology.

**Table 4:** Univariate and multivariate analyses to identify the independent correlation between high Lp(a) and high risk of PCa.

	Univariate model		Multivariate model	
	OR (95% CI)	*p* value	OR (95% CI)	*p* value
TG (mmol/l)	1.154 (0.700-1.902)	0.575		
TC (mmol/l)	0.931 (0.694-1.249)	0.633		
LDL-C (mmol/l)	0.839 (0.578-1.220)	0.358		
HDL-C (mmol/l)	1.196 (0.460-3.110)	0.714		
TC/HDL-C	0.818 (0.596-1.122)	0.213		
LDL-C/HDL-C	0.738 (0.493-1.106)	0.141		
RC (mmol/l)	1.054 (0.451-2.465)	0.903		
Lp(a) (mg/l)				
Quartile 1	1		1	
Quartile 2	1.864 (0.833-4.170)	0.130	1.957 (0.862-4.442)	0.109
Quartile 3	1.219 (0.584-2.545)	0.598	1.239 (0.581-2.641)	0.579
Quartile 4	2.687 (1.113-6.491)	**0.028**	2.890 (1.148-7.274)	**0.024**

Logistic regression analyses were performed. The multivariate model was adjusted for age, BMI, hypertension, diabetes, coronary artery disease, and lipid-lowering drugs. The bold value indicated statistical significance. PCa = prostate cancer; RC = remnant cholesterol; TC = total cholesterol; HDL-C = high-density lipoprotein cholesterol; LDL-C = low-density lipoprotein cholesterol; TG = triglycerides; Lp(a) = lipoprotein(a).

## Data Availability

The raw data that was used in this study is available upon request from the corresponding authors.
